# Circadian disruption induced by light-at-night accelerates aging and promotes tumorigenesis in rats

**DOI:** 10.18632/aging.100092

**Published:** 2009-10-02

**Authors:** Irina A. Vinogradova, Vladimir N. Anisimov, Andrey V. Bukalev, Anna V. Semenchenko, Mark A. Zabezhinski

**Affiliations:** ^1^ Petrozavodsk State University, pr. Lenina, 33, Petrozavodsk 185910, Russia; ^2^ Department of Carcinogenesis and Oncogerontology, N.N.Petrov Research Institute of Oncology, Pesochny-2, St.Petersburg 197748, Russia

**Keywords:** light-at-night, life span, tumorigenesis, rats

## Abstract

We evaluated the effect of various light/dark regimens on the survival, life span and tumorigenesis in rats.  Two hundred eight male and 203 females LIO rats were subdivided into 4 groups and kept at various light/dark regimens: standard 12:12 light/dark (LD); natural lighting of the North-West of Russia (NL); constant light (LL), and constant darkness (DD) since the age of 25 days until natural death. We found that exposure to NL and LL regimens accelerated development of metabolic syndrome and spontaneous tumorigenesis, shortened life span both in male and females rats as compared to the standard LD regimen. We conclude that circadian disruption induced by light-at-night accelerates aging and promotes tumorigenesis in rats. This observation supports the conclusion of the International Agency Research on Cancer that shift-work that involves circadian disruption is probably carcinogenic to humans.

## Introduction

The alternation of the day and night circadian cycle is a most important regulator of a wide variety of physiological rhythms in living organisms, including humans [1,2].   Due to the introduction of electricity and artificial light about hundred years ago the pattern and duration of human exposure to light has changed dramatically, and thus light-at-night has become an increasing and essential part of modern lifestyle. Light exposure at night seems to be associated with a number of both serious behavioral and health problems, including excess of body mass index, cardiovascular diseases, diabetes and cancer [3-14]. On the basis of  "limited evidence in humans for the carcinogenicity of shift-work that involves night work", and "sufficient evidence in experimental animals for the carcinogenicity of light during the daily dark period (biological night)"  the International Agency for Research on Cancer (IARC) Working Group concluded that "shift-work that involves circadian disruption is probably carcinogenic to humans" (Group 2A) [15].

Erren and Pekarski [16] suggested that indigenous populations in the Arctic region should be at lower risk of cancer. Cancer incidence in the Sami living in the far north of Europe have reported a lower risk than expected [17-19]. It is worth to note that mortality among Alaskan native peoples (Eskimo, Indian and Aleut) from breast cancer has tripled since 1969 for unknown reason [20]. We believe that an increase in light pollution could be one of causes of this phenomenon.

In the special issue of the International Journal of Circumpolar Health (December 2008; 67:5), the data on cancer incidence in circumpolar populations have been presented [21-25]. It was stressed that there is no consistent pattern of the cancer risk level among circumpolar indigenous people relative to European or North American populations [26]. The role of genetic diversion and life style as well as methodological differences in approaches to extract ethnic-specific data should be evaluated for solution of the problem [26]. Analysis of the data on cancer risk presented in the "Cancer in Five Continents" published by IARC has shown that there is a significant positive correlation between geographical latitude and the incidence of breast, colon and endometrial carcinomas and absence of the correlation in a case of stomach and lung cancers [27].

According to the circadian disruption hypothesis, light-at-night might disrupt the endogenous circadian rhythm, and specifically suppress nocturnal production of pineal hormone melatonin and its secretion in the blood [13-15]. However, a number of other mechanisms in addition to melatonin suppression can be involved into the process of development of pathologies at the constant illumination. Moreover, there are no available data on effect of natural light/dark regimen at circumpolar region on life span and tumorigenesis in rodents.  The aim of this study was to evaluate the effect of various light/dark regimens on some parameters of  homeostasis and of biological age, survival, life span and tumorigenesis in male and female rats.

**Figure 1.**
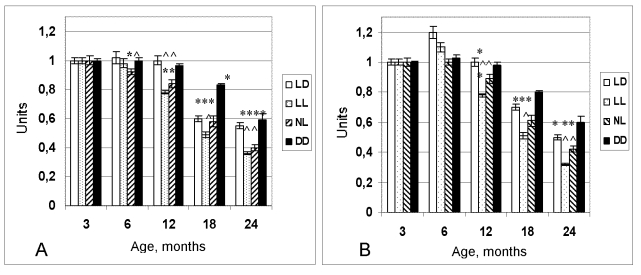
Dynamics of the coefficient of homeostatic stability (CHS) in female (**A**) and male (**B**) rats maintained at various light regimens. ^ The difference with the relevant parameter in the group LD is significant, р<0,05; * The difference with the parameter at the age of 3 months in the same group is significant, р<0,05 (Wilcoxon-Mann-Whytney test).

## Results

### Effect of light/dark regimen on homeostatic parameters in rats

Age-related body weight gain followed by its decrease was observed in rats of all groups at any light/dark regimens. However maximal weight of rats maintained at the LD or NL regimens was observed at the age of 15 months, whereas in the animals kept at the LL - at the 12th month. The number of rats with abdominal obesity was increased in the LL and NL groups as compared with the LD group (data are not shown).  Food consumption widely has been varying in all groups during the period of observation. There were periods of an increase in food consumption and those of a decrease. In general, in autumn and winter rats ate more lab food than in spring and summer. Male rats from the LL and NL groups ate more food compared with the LD group at the age of 18 and 21 months.

Monthly testing for glucosuria showed that there were no such cases until the age of 16 months in all groups. At the age of 16 months, 20% of rats from the group maintained at the LL regimen had glucose in urine, whereas at the age of 24 months 60% of rats in his group had glucosuria. In the NL group 40% of rats had glucosuria at the age of 18 months. Both serum glucose level and that of serum C-peptide were much higher in the LL and NL rats at the age of 18 and 24 months compared with the LD rats.

**Figure 2.**
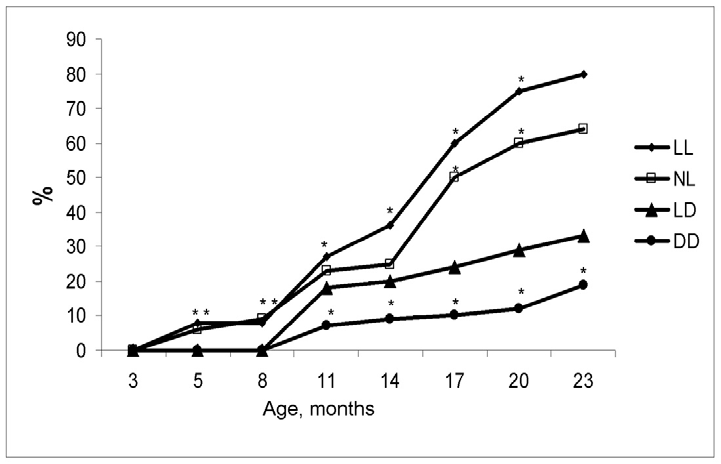
Age-related dynamics of incidence of irregular estrous cycles in rats maintained at various light/dark  regimens. Ordinate, number of rats with irregular estrous cycles (%). The difference with the relevant parameter in the group LD is significant, р<0.05.

The level of the serum cholesterol and β-lipoproteins was higher in young 3-months old rats and significantly decreased at the age of 6 months. Hence age-related increase of serum cholesterol and β-lipoproteins levels was observed to take place in rats of all groups. It worthy of note, that the level of β-lipoproteins was higher in the LL and NL rats compared with the LD rats at the age of 18 and 24 months.

At the age of 6 months the coefficient of homeostatic stability (CHS) was practically same in all groups. Age-related decrease of the CHS was observed in all groups as well. However most significant decline of its value has been observed in the groups NL and LL.  At the age from 12 to 24 months CHS in these groups was significantly lower in comparison to these in the LD group (Figure 1).  In females, age-related increase in the number of rats with irregular estrous cycles was accelerated both in the NL and the LL groups whereas was postponed in the DD group (Figure 2). Thus, constant and natural illumination accelerated aging in rats evaluated by age-related dynamics of CHS.

### Effect of light/dark regimen on life span in rats

In male rats, the exposure to both NL and LL regimens failed significantly influence the mean life span of all as well as the last of 10% survivors (Table 1).

**Figure 3.**
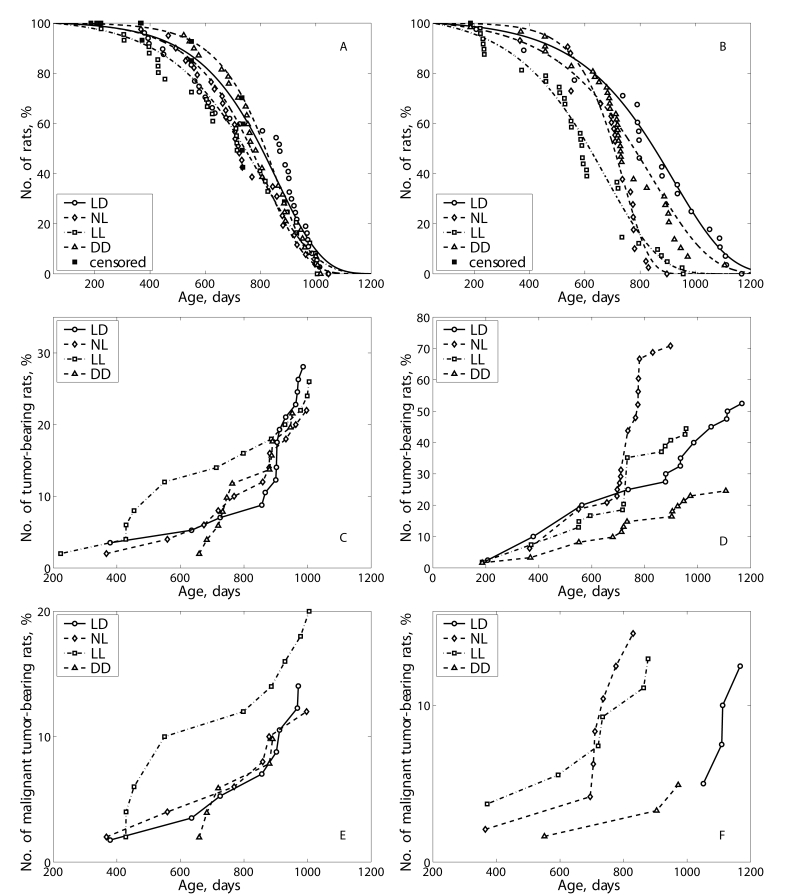
Effect of the exposure to various light regimens on survival and tumorigenesis in rats. (**A**) - survival, males; (**B**)- survival- females; (**C**) - total tumor incidence, males; (**D**) - total tumor incidence, females; (E) - malignant tumor incidence, males; (**F**) - malignant tumorincidence, females.

At the same time, the rate of population aging (parameter α in the Gompertz equation)  was slightly increased in NL group and decreased in LL as compared with the LD group. The survival curves for groups NL and LL were significantly shifted to left in comparison to the survival curve for the group LD (Figure 3A). The log-rank test shows the significant difference in the distribution of survivors between groups LD and NL (р= 0.001; χ^2 ^= 10.3) and between groups LD and LL (р= 0.01; χ^2 ^= 6.7). In ANOVA test the dependence of the life span on light regimen has been significant (15.45%; F=15.32; p<0.001). Thus, both LL and NL regimens accelerated mortality in male rats.

In female rats, the exposure to both NL and DD regimens failed significantly influence the mean life span of all as well as the last of 10% survivors, however the exposure to the LL regimen significantly decreased the life span (Table 2). At the same time, the rate of population aging was significantly increased by 2.1 times in the NL group and, correspondingly, decreased  the MRDT as compared with the LD group. The survival curves for groups NL and DD were significantly shifted to left in comparison to the survival curve for the group LD  (Figure 3B). The log-rank test shows the significant difference in the distribution of survivors between groups LD and NL (р= 0.0000243; χ^2 ^=22.2), between the groups LD and LL (p=0.0000162; χ^2 ^= 23.0) and between LD and DD (p=0.0741; χ^2 ^=3.2). In ANOVA test the dependence of the life span on light regimen has been significant (15.45%; F=15.32; p<0.001). Thus, both LL and NL regimens accelerated mortality in female rats.

**Table 1.** Effect of light regimen on survival and life span in male rats. Effect of light/dark regimen on spontaneous tumorigenesis in rats. Notes: Difference with controls is significant: a, р<0.05. #, in brackets 95% confidential intervals. MRDT, mortality rate doubling time.

Parameters	Light/dark regimen			
LD	NL	LL	DD
Number of rats	57	50	50	51
Mean life span, days	644 ± 34.0	613 ± 32.9	580 ± 35.5	652 ± 32.5
Maximum life span, days,	1045	1046	1005	1017
Mean life span of last 10% survivals, Days	999 ± 11.5	972 ± 22.7	983 ± 13.8	987 ± 13.0
α x 10^3^, days^-1^	6.06 (5.87; 6.47)	6.70^a^ (6.50; 6.97)	5.19^a^ (4.89; 5.57)	8.31^a^ (8.10; 8.54)
MRDT, days	112.4 (107.1; 118.1)#	103.4 (102.7; 111.6)	133.6^a^ (124.4; 141.7)	83.4^a^ (81.2; 85.5)

**Table 2.** Effect of light regimen on survival and life span in female rats. Notes: Difference with controls is significant: a, р<0.05; b, р<0.01; c, р<0.001. #, in brackets 95% confidential intervals. MRDT, mortality rate doubling time.

Parameters	Light/dark regimen			
LD	NL	LL	DD
Number of rats	40	48	54	61
Mean life span, days	706 ± 46.2	611 ± 29.5	526 ± 30.4^b^	639 ± 30.1
Maximum life span, days,	1167	897	956	1266
Mean life span of last 10% survials, days	1119 ± 16.7	830 ± 18.9	909 ± 19.1^c^	1023 ± 56.0
α x 10^3^, days^-1^	5.00 (4.73; 5.30)#	10.5 (10.3; 11.2)^a^	5.21 (5.13; 5.35)	4.88 (4.86; 4.97)
MRDT, days	138.6 (130.8; 146.6)	65.8 (61.7; 67.0)^a^	133.1 (129.6; 135.1)	142.0 (139.4; 142.5)

###  Effect of light/dark regimen on spontaneous tumorigenesis  in rats

Pathomorphological analysis shows that benign tumors were most frequent in all groups of males. The significant part of them was represented by testicular Leydig cell tumors (Table 3).  Among malignant tumors lymphomas were most common however some cases of hepatocellular carcinoma, soft tissues sarcomas and sporadic carcinomas were detected.

The exposure to the LL regimen accelerated spontaneous tumors development as compared to the LD group and not influenced their incidence in male rats (Table 3; Figure 3C). The first tumor in the LL group was detected 5 months earlier, and the first tumor in the DD group was observed 9 months later than the first tumor in the LD group.

In the female groups NL, the total incidence of tumors was significantly increase as compared with the LD group mainly due to practically 2-times increase in incidence of benign mammary tumors. It worthy of note that in the NL group 3 endometrial adenocarcinomas have been observed whereas no such type of malignancies where revealed in the LD group.  The light deprivation (group DD) significantly inhibited the development of all tumors, mainly mammary neoplasia.  The index of tumor multiplicity (number of tumors per tumor-bearing rat) was maximal (1.63) in the group LL and minimal (1.07) in the group DD (Table 4; Figure 3D).

**Table 3.** Effect of light regimen on tumorigenesis in male rats. Notes:  TBR - tumor-bearing rats.

**Parameters**		**Light/dark regimen**				
**LD**	**NL**		**LL**	**DD**
Number of rats		57	50		50	51
Number of TBR (%)		17 ( 29.8 %)	11 (22%)		13 (26%)	11 (21.6%)
No. of tumors per TBR		1.35	1.18		1.08	1.36
Number of malignant TBR (%)		7 (12.3%)	6 (12%)		10 (20%)	5 (9.8%)
Total number of tumors		23	13		14	15
Time of the 1^st^ tumor detection, days		379	367		223	659
Mean life span of TBR, days		824 ± 49.0	782 ± 57.6		688 ± 73.2	805 ± 32,3
Mean life span of malignant TBR, days		794 ± 72.4	738 ± 95.6		701 ± 76.0	766 ± 49.5
*Localization and type of tumors*						
Testes: Leydigoma hemangioma	7		6	4		6
	1		-	-		-
Malignant lymphoma/ leukemia	3		4	6		3
Liver: hepatocarcinoma	2		-	2		-
Skin: papilloma	1		-	-		-
Soft tisusues: fibroma sarcoma malignant fibrous histiocytoma	-		-	-		1
	1		2	-		1
	2		-	-		-
Lung: adenocarcinoma light-cell carcinoma	-		-	1		-
	1		-	-		-
Small bowel: adenocarcinoma	-		-	1		-
Adrenal gland: cortical adenoma pheochromocytoma malignant pheochromocytoma	3		1	-		3
	1		-	-		-
	-		-	-		1
Urether: fibroma	1		-	-		
Total: benign malignant	14		7	4		10
	9		6	10		5

## Discussion

Thus our data have shown that live-long maintenance of male and female rats at the NL or LL regimens accelerated age-related changes evaluated by the CHS, decreased life span and promoted spontaneous tumorigenesis. These data and some additional results of this study reported earlier are summarized in the Table 5 and supported this conclusion.

It was reported that risk of cancer is low in indigenous populations in Arctic [17-19]. However there are data on significant increase in the breast carcinoma risk in them since 1969 [20]. The cause of this phenomenon is unknown. The one of the reason could be the increase in light pollution.  Experiments in female rodent presented significantly evidence that exposure to constant illumination (24 hours per day) leads to disturbances in estrus function (persistent estrus syndrome, anovulation) [31,36,37] and spontaneous tumor development [4,34,36,38]. The evidence of promoting effect of exposure to constant illumination on mammary carcinogenesis induced by chemical carcinogens are discussed elsewhere [3,4,14]. This paper in the first time has shown that the  exposure of male rats  to the constant illumination accelerated the development spontaneous tumors. This paper firstly have shown that maintenance of female rats to natural light conditions of the north (long "white night" and "polar night" seasons) also leads to premature switching-off of reproductive function and promotion of spontaneous carcinogenesis.

**Table 4.** Effect of light regimen on tumorigenesis in female rats. Notes:  TBR -  tumor-bearing rats. Difference with the group LD is significant: a, р<0.05; b, р<0.01; c, р<0.001.

**Parameters**	**Light/dark regimen**				
**LD**		**NL**	**LL**	**DD**
Number of rats	40		48	54	61
Number of TBR (%)	21 (52.5%)		34 (70.8%)^a^	24 (44.4%)	15 (24.6%)^c^
No. of tumors per TBR rat	1.38		1.41	1.63	1.07
No. of mlgn. TBR rats (%)	5 (12.5%)		7 (14.6%)	7 (13.0%)	3 (4.9%)
Number of tumors	29		48	39	16
Time of the 1^st^ tumor detection, days	207		365	186	186
Mean life span of TBR, days	769 ± 63.0		683 ± 22.9^b^	665 ± 40.3	720 ± 64.3
Mean life span of malignant TBR, days	1098 ± 21.8		688 ± 56.8^c^	647 ± 79.8^c^	809 ± 130.5^a^
*Localization and type of tumors*					
Mammary gland: fibroma fibroadenoma adenocarcinoma		4	9	1	-
		11	21	20	5
		-	-	1	-
No. of rats with benign mammary tumors		14	27^a^	18	5^c^
Utery: polyp fibroma fibromyoma adenocarcinoma stromogenic sarcoma		4	1	4	5
		1	-	1	-
		-	2	1	-
		-	3	1	-
		-	-	-	1
Oviduct: fibroma		-	3	-	-
Adrenal gland: cortical adenoma carcinoma pheochromocytoma		1	1	3	1
		-	-	-	1
		2	-	-	-
Ovary: fibroma luteoma hemangioma carcinoma		1	-	-	-
		-	1	-	-
		-	-	-	1
		-	1	-	-
Pituitary: adenoma		-	-	1	-
Hematopoeitic tissue: leukemia/lymphoma		3	3	4	-
Soft tissues: fibroma sarcoma		-	1	1	1
		2	2	1	-
Lung: adenocarcinoma		-	-	-	1
Colon: adenocarcinoma		-	1	-	-
Total: benign malignant		24	39	32	13
		5	9	7	3

**Table 5.** Summary evaluation of effects of various light/dark regimen on biomarker of aging and homeostatic parameters in female and male rats [ 28-35]. Notes:  ↑ - increases (acceleration); ↓ - decreases (slow down); = - no effect, as compared with the parameter in the LD group; M - male; F - female.

Parmeters	Sex	Ligh/dark regimen					
NL			LL		DD
Body weight	Male	↑			=		=
	Female	↓			↓		↓
Body weight gain	M & F	↓			↓		↑
Progressive growth period	Male	↑			↓		↑
Stable growth period	Male	↓			↓		↓
Presenile period	Male	↑			↑		↑
Senile period onset	Male	↑			↑		↓
Food consumption	M & F	↑			↑		↓
Water consumption	Male	=			=		=
Maturity onset	M & F	↑			↑		↓
Estrous function switching-off	Female	↑			↑		↓
Diuresis	Male	↓			↓		↓
Morbidity	M & F	↑			↑		↓
*Biochemical parameters in urine*							
Glucose (age at appearance)	Male	↑		↑			↓
Ketones (age at appearance)	Male	↑		↑			↑
*Behavioral, cognitive and physical activity*							
Locomotor activity	Male	↑		↑		↓	
Psychoemotional feautures	Male	↑		↑		↓	
Cognitive function	Male	↓		↓		=	
Dynamic endurance	Male	↓		↓		↑	
Static endurance	Male	↓		↓		↓	
*Biochemical parameters in blood*							
Glucose	M & F	↑	↑				=
Cholesterol	M & F	↑	↑				=
Β-lipoproteins	M & F	↑	↑				=
Total protein	M & F	↓	↓				↑
Urea	M & F	↑	↑				=
Creatinine	M & F	↑	↑				=
Sodium	M & F	=	=				=
Potassium	M & F	=	↓				↑
*Serum level of hormones *							
Prolactin	M & F	↑	↑				↓
C-peptide	M & F	↑	↑				=
TSH	M & F	↓	↓				=
Т4	M & F	↑	↑				=
T3	M & F	↓	=				↑
Coefficient of homeo-static stability (CHS)	M & F	↓	↓				↑
*Antioxidant system*							
Liver	M & F	↓		=		=	
Kidney	M & F	=		↓		=	
Heart	M & F	↓		↓		=	
Skeletal muscles	M & F	↓		↓		=	
Life span	Male	=		=		=	
	Female	↓		↓		↑	
Spontaneous carcinogenesis	M & F	↑		↑		↓	

The important finding of our experiments was an observation of manifestations of metabolic syndrome in rats kept at the NL and LL regimens. There is evidence of relationship between the pineal gland and physiological regulation of carbohydrate and lipid metabolism. Thus, in pinealectomized rats the decrease of tolerance to glucose, the increase in the level of total lipids, free fatty acids, disturbances in the ratio of free and bounded insulin were observed [39]. In patients with cardiac metabolic syndrome, lower nocturnal peak and Δ melatonin (peak - lowest melatonin level) were observed compared with normal healthy subjects [40]. Some epidemiological studies show that night-shift workers, whose activity period is chronically reversed, show an increased incidence of the metabolic syndrome [41]. It was demonstrated impaired glucose metabolism in mice with clock genes *Bmal1* or *Clock* mutations [42]. In homozygous mice with mutation in circadian *clock* gene the metabolic syndrome characterized by obesity, hyperlipidemia, hyperleptinemia, liver steatosis, hyperglycemia and hyperinsulinemia developed [43].  In our experiment, the rats exposed to the disturbed light/dark regimen developed the metabolic disorders which might be evaluated as a metabolic syndrome: abdominal obesity, hyper-cholesterolemia, hyperglycemia, hyperbetalipemia and glucosuria. It is worthy to note, that the life span was shorter and the incidence of spontaneous tumors was higher in the rats exposed to the LL or NL regimens compared the rats to be maintained at the standard LD regimen.  Chronic circadian disruption induced by chronic reversal in the light/dark cycle was followed by the reduction by 11% in the mean life span in cardiomyopathy-prone Syrian hamsters [44].

The insulin/insulin-like growth factor-1 (IGF-1) signaling pathway plays a fundamental role in animal physiology, influencing longevity, reproduction, and diapause in many species [45]. Despite a large number of studies, the role of melatonin on glucose metabolism is rather controversial [46-48].

Metabolic syndrome [49-51] characterized by obesity, hypertriglyceridemia and hypercholesterolemia, by decrease in the level of high density lipoproteins and blood fibrinolytic activity, by arterial hypertension, by lowering of tolerance to glucose and by rise in insulin resistance. The metabolic syndrome is a risk factor not only for cardiovascular diseases but for cancer too [45,51,52]. The inhibition of pineal function due to exposure to continuous light at night probably facilitates the metabolic syndrome development.

Thus our results shown that the natural light/dark regimen in Arctic as well as constant illumination acce-lerate the aging and increase the tumor incidence in rodents. The significance of these findings for human should be evaluated in well controlled population studies in humans.

### Material and methods

Two hundred eight male and 203 female outbreed LIO rats [53] were born during the first half of May, 2003. At the age of 25 days they were randomly subdivides into 4 groups (males and females separately) and kept at 4 different light/dark regimens: 1) standard alternating regimen (LD) - 12 hours light (750 lux): 12 hours dark; 2) natural light/dark regimen (NL) at the latitude of Petrozavodsk (N 61º47'') - in winter minimal lighting was 4.5 hours (polar night), in summer - 24 hours light on ("white nights"); illumination at the level of cages varied from 50 to 200 lux in the morning to 1000 lx for bright sunny day and about 500 lx for cloudy or rainy day;  3) constant light regimen (LL) -  24 hours light on (750 lux); 4) constant darkness (DD) - only dim red light (0 - 0.5 lux) was switching-on for animal service.

All animals were kept in the standard polypropylene cages at the temperature 21-23 ºC and were given *ad libitum* standard laboratory meal [54] and tap water. The study was carried out according to the recommendations of the Committee on Animal Research of Petrozavodsk State University about the humane treatment of animals.

All rats were weighted once a month and the amount of food consumed was measured. Two hundred grams of food were given in each cage after cleaning and 24 h later the food which was not eaten was collected from each cage and weighted. The mean amount of food (grams) consumed per rat for this day was calculated for each group.  Every month rats were placed into individual metabolic cages for urine collection. The concentration of glucose in the urine was estimated with Ames test system for urine ("Bayer", Germany).

Once every 3 months, daily for 2 weeks vaginal smears were cytologically examined in females to determine estrous function. At the age of  3, 6, 12, 18 and 24 months 10 male rats from each group were given guillotine after 24-hours fasting. Blood samples were taken in each animal. The collected samples were centrifuged and the serum was stored at -70 ºC for subsequent biochemical study. The serum level of free triiodothyronine (T3), thyroxin (T4) and thyroid stimulating hormone (TSH) was estimated by immunoenzymatic method (kits "Immulite"),  level of C-peptide and prolactin - by kits "BiochimMac", glucose - by enzymatic method, concentration of β-liporoteins - by turbidimetric method, cholesterol - with kits "Vital Diagnostics SPb", creatinine - with kits "Olvex Diagnosticum", urea - with kits "Abris+". Concentration of potassium and sodium ions was estimated by ionoselective method with ionometer ETs-59 (Russia).

Integral dynamics of age-related changes of studied biochemical parameters was evaluated as a Coefficient of Homeostatic Stability (CHS), which was estimated as a ratio of total number of biochemical and endocrine parameters equal to relevant their indices at the age 3 months to total number of parameters studied [55].

All other rats were allowed to survive for natural death. All animals were autopsied. Tumors as well as the tissues and organs with suspected tumor development were excised and fixed in 10% neutral formalin. After the routine histological processing the tissues were embedded into paraffin. 5-7μm thin histological sections were stained with hematoxylin and eosin and examined microscopically. Tumors were classified according to the IARC recommendations [56,57].

Experimental results were statistically processed by the methods of variation statistics with the use of STATGRAPH statistic program kit. The significance of the discrepancies was defined according to the Student *t*-criterion, Fischer exact method, χ^2^, non-parametric Wilcoxon-Mann-Whitney and Friedman RM ANOVA on Ranks. Student-Newman-Keuls method was used for all pairwise multiple comparisons. Coefficient of correlation was estimated by Spearman method [58]. Differences in tumor incidence were evaluated by the Mantel-Haenszel log-rank test.

Parameters of Gompertz model were estimated using maximum likelihood method, non-linear optimization procedure [59] and self-written code in 'Matlab'; confidence intervals for the parameters were obtained using the bootstrap method [60].

For experimental group Cox regression model [61] was used to estimate relative risk of death and tumor development under the treatment compared to the control group: h(t, z) = h_0_(t) exp(zβ), where h(t,z) and h_0_(t) denote the conditional hazard and baseline hazard rates, respectively,  β is the unknown parameter for treatment group, and z takes values 0 and 1, being an indicator variable for two samples − the control and treatment group. Semiparametric model of heterogeneous mortality [62] was used to estimate the influence of the treatment on frailty distribution and baseline hazard.
